# Impact of nutrition route on microaspiration in critically ill patients with shock: a planned ancillary study of the NUTRIREA-2 trial

**DOI:** 10.1186/s13054-019-2403-z

**Published:** 2019-04-05

**Authors:** Saad Nseir, Amélie Le Gouge, Jean-Baptiste Lascarrou, Jean-Claude Lacherade, Emmanuelle Jaillette, Jean-Paul Mira, Emmanuelle Mercier, Pierre-Louis Declercq, Michel Sirodot, Gaël Piton, François Tinturier, Elisabeth Coupez, Stéphane Gaudry, Michel Djibré, Didier Thevenin, Jeremy Pasco, Malika Balduyck, Farid Zerimech, Jean Reignier

**Affiliations:** 1 Médecine Intensive Réanimation, CHU Lille, F-59000 Lille, France; 2 Faculté de Médicine, Université de Lille, F-59000 Lille, France; 3 Inserm CIC 1415, Tours, France; 4 Université de Tours, Tours, France; 5 CHU Tours, Tours, France; 6 Médecine Intensive Réanimation, CHU de Nantes, Nantes, France; 7 Université de Nantes, Nantes, France; 8 Médecine Intensive Réanimation, Centre Hospitalier Départemental de la Vendée, La Roche-sur-Yon, France; 9 Medical Intensive Care Unit, Cochin University Hospital, Assistance Publique-Hôpitaux de Paris (AP-HP), Paris, France; 10 Médecine Intensive Réanimation, Hôpital Bretonneau, CHU Tours, Tours, France; 11 Médecine Intensive Réanimation, Hôpital de Dieppe, Dieppe, France; 12 Medical-Surgical Intensive Care Unit, Centre Hospitalier Annecy-Genevois, Metz-Tessy, Pringy France; 13 Medical Intensive Care Unit, CHRU Besançon, Besançon, France; 14 EA3920, Université de Franche Comté, Besançon, France; 15 Surgical Intensive Care Unit, CHU Amiens Picardie, Amiens, France; 16 Intensive Care Unit, Hôpital Gabriel Montpied, CHU de Clermont-Ferrand, Clermont-Ferrand, France; 17 Service de Réanimation Médico-Chirurgicale, Hôpital Avicenne, Assistance Publique-Hôpitaux de Paris (AP-HP), Bobigny, France; 18 INSERM, UMR_S1155, Remodeling and Repair of Renal Tissue, Hôpital Tenon, Paris, Paris, France; 19 Medical-Surgical Intensive Care Unit, Tenon University Hospital, Assistance Publique-Hôpitaux de Paris (AP-HP), Paris, France; 20 Medical-Surgical Intensive Care Unit, Centre Hospitalier Docteur Schaffner, Lens, France; 21 Centre de Biologie Pathologie, CHU Lille, F-59000 Lille, France; 22 Faculté de Pharmacie, Université de Lille, F-59000 Lille, France

**Keywords:** Microaspiration, Gastric contents, Oropharyngeal secretions, Pneumonia, Pathophysiology, Critical care

## Abstract

**Background:**

Microaspiration of gastric and oropharyngeal secretions is the main mechanism of entry of bacteria into the lower respiratory tract in intubated critically ill patients. The aim of this study is to determine the impact of enteral nutrition, as compared with parenteral nutrition, on abundant microaspiration of gastric contents and oropharyngeal secretions.

**Methods:**

Planned ancillary study of the randomized controlled multicenter NUTRIREA2 trial. Patients with shock receiving invasive mechanical ventilation were randomized to receive early enteral or parenteral nutrition. All tracheal aspirates were collected during the 48 h following randomization. Abundant microaspiration of gastric contents and oropharyngeal secretions was defined as the presence of significant levels of pepsin (> 200 ng/ml) and salivary amylase (> 1685 UI/ml) in > 30% of tracheal aspirates.

**Results:**

A total of 151 patients were included (78 and 73 patients in enteral and parenteral nutrition groups, respectively), and 1074 tracheal aspirates were quantitatively analyzed for pepsin and amylase. Although vomiting rate was significantly higher (31% vs 15%, *p* = 0.016), constipation rate was significantly lower (6% vs 21%, *p* = 0.010) in patients with enteral than in patients with parenteral nutrition. No significant difference was found regarding other patient characteristics. The percentage of patients with abundant microaspiration of gastric contents was significantly lower in enteral than in parenteral nutrition groups (14% vs 36%, *p* = 0.004; unadjusted OR 0.80 (95% CI 0.69, 0.93), adjusted OR 0.79 (0.76, 0.94)). The percentage of patients with abundant microaspiration of oropharyngeal secretions was significantly higher in enteral than in parenteral nutrition groups (74% vs 54%, *p* = 0.026; unadjusted OR 1.21 (95% CI 1.03, 1.44), adjusted OR 1.23 (1.01, 1.48)). No significant difference was found in percentage of patients with ventilator-associated pneumonia between enteral (8%) and parenteral (10%) nutrition groups (HR 0.78 (0.26, 2.28)).

**Conclusions:**

Our results suggest that enteral and parenteral nutrition are associated with high rates of microaspiration, although oropharyngeal microaspiration was more common with enteral nutrition and gastric microaspiration was more common with parenteral nutrition.

**Trial registration:**

ClinicalTrials.gov, NCT03411447. Registered 18 July 2017. Retrospectively registered.

## Background

Microaspiration of contaminated oropharyngeal and gastric secretions is main route of entry of bacteria into the lower respiratory tract in patients receiving invasive mechanical ventilation [1, 2]. The incidence of microaspiration is high in critically ill intubated patients, ranging 20–60% [3–5]. Risk factors for microaspiration are related to tracheal tube, mechanical ventilation, nutrition, and patient-specific factors [6]. Although tracheobronchial colonization is common in critically ill patients, only a small proportion of patients develop subsequent ventilator-associated pneumonia (VAP) [7]. This infection is associated with increased morbidity, mortality, and cost [8].

Enteral nutrition is associated with increased risk of vomiting [9]. Studies highlighted the fact that enteral nutrition might increase proliferation of bacteria in the stomach. The relationship between enteral nutrition, aspiration, and VAP has been investigated during the last decades. Although some studies identified enteral nutrition as a risk factor for VAP [10, 11], other large well-conducted studies did not confirm this finding [12, 13]. Prevention of vomiting with monitoring of gastric residual volume in mechanically ventilated patients receiving early enteral feeding was not associated with decreased risk of VAP [12]. In the NUTRIREA2 trial on the route of early nutritional support in mechanically ventilated patients with shock, early enteral nutrition was associated with more vomiting but not with increased rates of VAP, compared to early parenteral nutrition [13]. However, to our knowledge, no study to date has specifically evaluated the impact of the route of feeding on microaspiration of gastric contents and oropharyngeal secretions. Better understanding of pathophysiology of microaspiration and VAP could be helpful to improve preventive strategies for this infection. For this purpose, we planned an ancillary study of the NUTRIREA2 trial to determine the impact of enteral nutrition, compared with parenteral nutrition, on abundant microaspiration of gastric contents and oropharyngeal secretions. Our main hypothesis was that early enteral nutrition, compared to parenteral, might increase the risk of microaspiration of gastric contents in intubated critically ill patients.

## Methods

This was a planned ancillary study (ClinicalTrials.gov, identifier NCT03411447) of the randomized controlled multicenter open-label NUTRIREA2 study (ClinicalTrials.gov, identifier NCT01802099). The NUTRIREA-2 study was supported by the Programme Hospitalier de Recherche Clinique National 2012 of the French Ministry of Health (#PHRC-12-0184) and was designed to compare the effect of early enteral or parenteral nutrition on mortality in adult patients with shock requiring invasive mechanical ventilation [13, 14]. All centers participating to NUTRIREA2 study were invited to participate in the ancillary study, and 13 of them accepted to participate. All ICUs were French medical, surgical, or mixed ICUs.

The study protocol was approved by the ethics committee of the French Intensive Care Society and appropriate French authorities (Comité de Protection des Personnes de Poitiers) (CHD085-13). According to French law, because the treatments and strategies used in the study were classified as standard care, there was no requirement for signed consent, but the patients or next of kin were informed about the study before enrolment and confirmed this fact in writing.

### Study patients

Inclusion and exclusion criteria were similar to those of the NUTRIREA2 study. No additional criteria were required for the current study. In all participating ICUs, consecutive patients included in the NUTRIREA2 trial were included in the current ancillary study until the predetermined goal of sample size was met. Thus, adults (18 years or older) admitted to any of the participating ICUs were eligible if they were expected to require more than 48 h of invasive mechanical ventilation, concomitantly with vasoactive therapy (adrenaline, dobutamine, or noradrenaline) via a central venous catheter for shock, and to be started on nutritional support within 24 h after tracheal intubation (or within 24 h after ICU admission if intubation occurred before ICU admission). Exclusion criteria were invasive mechanical ventilation started more than 24 h earlier; surgery on the gastrointestinal tract within the past month; history of gastrectomy, oesophagectomy, duodeno-pancreatectomy, bypass surgery, gastric banding, or short bowel syndrome; gastrostomy or jejunostomy; specific nutritional needs, such as pre-existing long-term home enteral or parenteral nutrition; active gastrointestinal bleeding; treatment-limitation decisions; adult under legal guardianship; pregnancy; breastfeeding; current inclusion in a randomized trial designed to compare enteral nutrition to parenteral nutrition; contraindication to parenteral nutrition (known hypersensitivity to egg or soybean proteins or to another component, inborn error in aminoacid metabolism, or severe familial dyslipidemia affecting triglyceride levels). Further details on methods are available in the published protocol of the NUTRIREA2 study [14].

All study patients were positioned in semirecumbent position during their period of mechanical ventilation. Tracheal cuff pressure was monitored using a manometer and adjusted around 25 cmH_2_O, three times a day. After randomization and starting enteral or parenteral nutrition, all tracheal aspirates were collected for 48 h for pepsin and alpha amylase measurements. Tracheal aspirates were performed according to the need of each individual patients, as determined by nurses at bedside. Saline instillation was not recommended during sampling for tracheal aspirates. All tracheal aspirates were stored at − 20 °C and sent to the central laboratory at Lille University Hospital, where all measurements were blindly performed (ELISA technique for pepsin and difference between total and pancreatic amylase activity for salivary amylase).

### Definitions

Abundant microaspiration of gastric contents was defined by the presence of pepsin at significant concentration (> 200 ng/ml) in > 30% of tracheal aspirates. Abundant microaspiration of oropharyngeal secretions was defined by the presence of amylase at significant concentration (> 1685 IU/ml) in > 30% of tracheal aspirates [4, 15].

The primary outcome was percentage of patients with abundant microaspiration of gastric contents. Secondary outcomes were the percentage of patients with abundant microaspiration of oropharyngeal secretions, the percentage of tracheal aspirates positive for pepsin, the percentage of tracheal aspirates positive for alpha amylase, and the percentage of patients with VAP.

### Statistical analyses

We calculated that a sample of 188 patients (94 patients per group) would provide a power of 80% to detect an absolute risk reduction in primary endpoint of 20% in the parenteral nutrition group, with a two-sided type I error of 0.05, assuming a primary endpoint rate of 50% in the enteral nutrition group.

All analyses were performed in all randomized patients on the basis of their original group of randomization, according to the intention-to-treat principle. Qualitative variables were expressed as frequencies and percentages and compared using chi-square test. Quantitative variables were expressed as median (interquartile range) and compared between the two groups using Wilcoxon test. In order to take into account competition of some variables with the risk of death, survival analyses were performed using Fine and Gray’s method.

The rate of patients with abundant microaspiration of gastric contents, or abundant microaspiration of oropharyngeal secretions, was compared between study groups using chi-square, and OR (95% CI) was calculated. Additional adjusted analysis was performed, including variables collected at ICU admission with *p* < 0.1 in the multivariable logistic regression model.

Data were analyzed using SAS version 9.4 (SAS Institute, Cary, NC, USA) and R version 3.3.1.

## Results

**Fig. 1:**
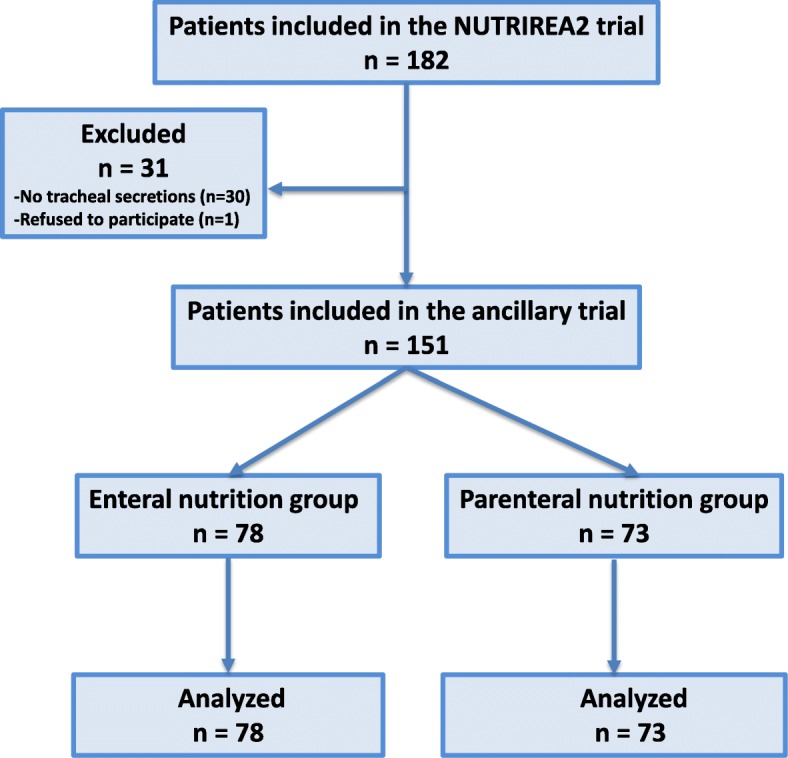
Study flowchart

One hundred eighty-two patients were included in the NUTRIREA2 study in the 13 centers participating in the ancillary study from February through June 2015, and 151 (84%) patients were included in the ancillary study. Among the 151 included and analyzed patients, 78 and 73 received enteral and parenteral nutrition, respectively (Fig. 1). The estimated sample size was not reached, because the NUTRIREA2 study was early stopped after the second interim analysis and because the ancillary study was only conducted during a 5-month period. One thousand seventy-four tracheal aspirates were quantitatively analyzed for pepsin and alpha amylase. The number of samples per patient was not significantly different between the two groups (median (IQR) 7 (4–10) vs 6 (4–9), *p* = 0.862, in enteral and parenteral nutrition groups, respectively).

### Patient characteristics

No significant difference was found in patient characteristics at ICU admission between the two groups (Table 1). Percentage of patients with vomiting and number of days with enteral nutrition was significantly higher in patients in the enteral nutrition group, compared to patients in parenteral nutrition group. Percentage of patients with constipation and number of days with parenteral nutrition were significantly higher in patients in the parenteral nutrition group, compared to patients in enteral nutrition group. No significant difference was found in other patient characteristic during ICU stay (Tables 2 and 3).

**Table 1:** Patient characteristics at ICU admission

	Enteral nutrition *n* = 78	Parenteral nutrition *n* = 73	*p* values
Male gender	59 (76)	55 (75)	0.966
Age	66.1 (56.4, 73.8)	64.5 (56.7, 75)	0.936
Body mass index	28 (24, 32.1)	27.7 (25.5, 33.4)	0.405
SOFA score	10 (9, 12.8)	11 (9, 13)	0.290
SAPS II	56 (47.3, 69.8)	58 (42, 72)	0.865
Prone position	6 (8)	6 (8)	0.905
Any chronic disease	54 (69)	48 (66)	0.648
Diabetes	20 (26)	16 (22)	0.592
Cause for ICU admission			0.688
Cardiac arrest	8 (10)	9 (12)	
Circulatory failure	14 (18)	9 (12)	
Neurologic failure	7 (9)	5 (7)	
Respiratory failure	39 (50)	34 (47)	
Trauma	1 (1)	2 (3)	
Others	9 (12)	14 (19)	
Category of admission			0.697
Medical	70 (90)	64 (88)	
Planned surgery	2 (3)	1 (1)	
Urgent surgery	6 (8)	8 (11)	
Treatment			
Insulin	30 (38)	27 (37)	0.852
Erythromycin or metoclopramide	1 (1)	1 (1)	0.962
Anti-H2 or proton pump inhibitor	28 (36)	32 (44)	0.319
Antibiotics	63 (81)	59 (81)	0.993
Sedation	63 (81)	59 (81)	0.993
Opioids	56 (72)	61 (84)	0.084
Neuromuscular-blocking agents	27 (35)	28 (38)	0.633
MacCabe score			0.772
No fatal disease	52 (67)	45 (62)	
Chronic fatal disease (in 5 years)	21 (27)	24 (33)	
Chronic fatal disease (in 1 year)	5 (6)	4 (5)	
Tracheal tube size	7.5 (7.5, 7.5)	7.50 (7, 7.5)	0.068

Results are *n* (%) or median (interquartile range)

**Table 2:** Patient characteristics after inclusion

	Enteral nutrition *n* = 78	Parenteral nutrition *n* = 73	*p* values
During the 48 h following inclusion			
Lowest tracheal cuff pressure, cmH_2_O	28 (20.5, 30)	28 (20, 30)	0.844
Highest tracheal cuff pressure, cmH_2_O	30 (30, 32)	30 (30, 30)	0.169
Lowest PEEP, cmH_2_O	5 (5, 6)	6 (5, 7)	0.137
Highest PEEP, cmH_2_O	8 (5, 12)	8 (6, 12)	0.150
Maximal norepinephrine dose, mg/h	1.6 (0.9, 3.2)	2.2 (1.41, 3.8)	0.062
During the first week of ICU stay			
Number of days with parenteral nutrition	0 (0, 0)	4 (4, 6)	< 0.001
Number of days with enteral nutrition	6 (4, 8)	1 (0; 4)	< 0.001
Daily calory intake (Kcal/kg/24 h)	19.1 (15.2, 21)	19.6 (16.7, 21.6)	0.257
Constipation at day 6	5 (6)	15 (21)	0.010
Other outcomes			
28-day mortality	32 (41)	20 (27)	0.078
90-day mortality	36 (46)	26 (36)	0.212
ICU length of ICU stay, days	9 (5.3, 16)	12 (7, 19)	0.148
Acute-care hospital length of stay (days)	16 (7, 31.8)	21 (12, 29)	0.182
Mechanical ventilation free days	12 (0, 22)	13 (0, 24)	0.102

Results are *n* (%) or median (interquartile range)

**Table 3:** Other patient characteristics (competitive risk analyses)

	Enteral nutrition *n* = 78	Parenteral nutrition *n* = 73	HR	*p* values
Vomiting	22	3	8.49 [1.99, 36.30]	0.004
Prokinetic drugs	15	3	5.78 [1.29, 25.90]	0.022
Stress ulcer prophylaxis	66	70	0.93 [0.71, 1.22]	0.590
Anti-infectious treatment	96	95	0.99 [0.87, 1.14]	0.940
Prone position	14	16	0.85 [0.39, 1.85]	0.680
Ventilator-associated pneumonia	8	10	0.78 [0.26, 2.28]	0.650

Results are percentages during the 48 h after randomization, except for ventilator-associated pneumonia (until day 28)

### Primary and secondary outcomes

**Fig. 2:**
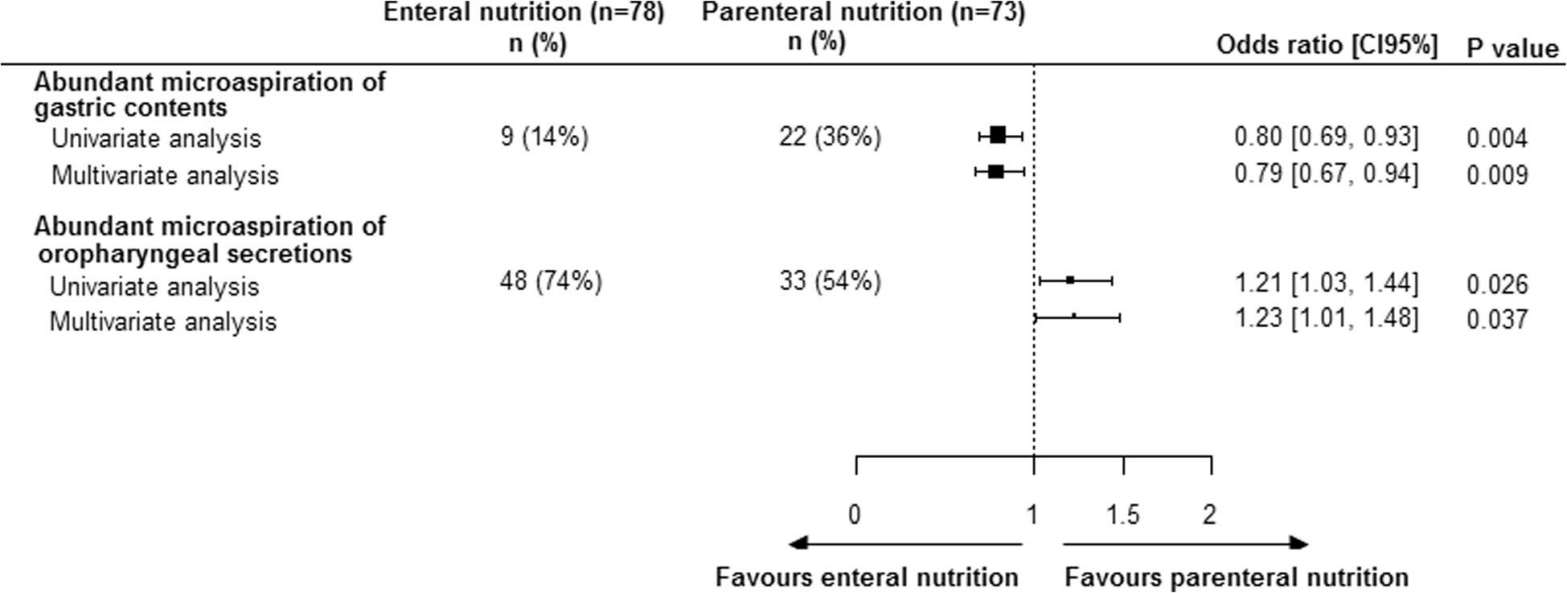
Impact of nutrition route on abundant microaspiration of gastric contents and oropharyngeal secretions. *Adjusting for tracheal tube size and opioid use at admission

The percentage of patients with abundant microaspiration of gastric contents was significantly lower in enteral nutrition group, as compared with parenteral nutrition group (9 of 78 (14%) vs 22 of 73 patients (36%), unadjusted OR 0.8 (95% CI 0.69–0.93), and adjusted OR (0.79 (0.67–0.94)). The percentage of patients with abundant microaspiration of oropharyngeal secretions was significantly higher in enteral nutrition group, as compared with parenteral nutrition group (48 of 78 (74%) vs 33 of 73 patients (54%), unadjusted OR 1.21 (95% CI 1.03–1.44), and adjusted OR 1.23 (1.01–1.48) (Fig. 2).

### Other outcomes

The median (IQR) percentage of tracheal aspirates positive for pepsin (0 (0, 11) vs 0 (0, 50), *p* = 0.044) was significantly lower in enteral, compared with parenteral nutrition groups. The median pepsin level in study patients (83 (24, 152) vs 97 (42, 228) ng/ml, *p* = 0.090) was not significantly different between enteral and parenteral nutrition groups. The median (IQR) percentage of tracheal aspirates positive for alpha amylase (75 (25, 100) vs 33 (0, 100), *p* = 0.055), and the median alpha amylase level in study patients (4283 (1707, 17,253) vs 1916 (644, 8419) IU/ml, *p* = 0.069) were not significantly different between enteral and parenteral nutrition groups, respectively.

No significant difference was found in pepsin level between patients with vomiting and those with no vomiting (median 157 (IQR 23, 278) vs 88 (34, 162) ng/ml, *p* = 0.34), or between patients who received stress ulcer prophylaxis and those who did not (median 89 (IQR 28, 164) vs 104 (43, 200) IU/ml, *p* = 0.35). No significant difference was found in salivary amylase level between patients with vomiting and those with no vomiting (median 8419 (IQR 1048, 72,365) vs 3038 (838, 17,036) IU/ml, *p* = 0.17).

## Discussion

To our knowledge, our study is the first to evaluate the relationship between the route of nutrition and the risk for microaspiration in intubated and mechanically ventilated patients, using valuable biomarkers of gastric and oropharyngeal secretion microaspiration. Our results suggest that enteral nutrition, as compared with parenteral nutrition, is associated with reduced risk for abundant microaspiration of gastric contents and increased risk for abundant microaspiration of oropharyngeal secretions.

The role of the stomach in the pathogenesis of VAP has been a matter for debate during the last decades. Although some studies suggested that the stomach played an important role in the occurrence of VAP [10, 11, 16], others did not identify the stomach as a source of microorganisms responsible for VAP [13, 17]. A study using molecular typing for all microorganisms coming from oropharyngeal, gastric secretions, tracheal aspirates, and bronchoalveolar lavage in patients with suspected VAP suggested that the stomach was the second source (17% of cases) for bacteria responsible for VAP, after the oropharynx (47% of cases) [18]. In addition, our group performed a randomized controlled study to determine the impact of continuous control of cuff pressure on abundant microaspiration of gastric contents and VAP [16]. This intervention significantly reduced both, suggesting a link between gastric content microaspiration and VAP. However, the results of the current study suggest that enteral nutrition, as compared with parenteral nutrition, is not associated with increased risk for microaspiration of gastric contents.

Some potential explanations could be suggested for this unexpected result. In patients receiving enteral nutrition, pepsin levels might have been artificially reduced because of pepsin dilution in the large quantity of liquid given for enteral nutrition. Further, enteral nutrition increases gastric pH [19] and could reduce the secretion of pepsin, as activation of pepsinogen into pepsin takes place in low pH. To our knowledge, no study specifically evaluated the effects of enteral nutrition, versus parenteral, on salivary amylase secretion. However, previous studies showed that enteral nutrition resulted in vagal afferent activation and parasympathetic stimulation, resulting in increased salivary secretion [20, 21]. In addition, a decreased production of digestive enzymes, including saliva, was reported in patients receiving exclusive parenteral nutrition [22]. Vasoactive drugs also impact the secretion of saliva [23]. However, no significant difference was found in the maximal dose of norepinephrine between the two groups.

Our results are in line with the NUTRIREA1 multicenter randomized trial, which showed that, compared to routine residual gastric volume (RGV) monitoring, absence of RGV monitoring in patients receiving invasive mechanical ventilation was associated with higher vomiting rate but no increased risk of VAP [12]. Thus, increased vomiting may not be associated with increased aspiration of gastric content in mechanically ventilated patients receiving enteral nutrition. Interestingly, measurements of amylase in the current study indicate increased aspiration of oropharyngeal secretions in patients with enteral nutrition, compared to those who received parenteral nutrition. However, no significant difference was found in VAP rates between the two groups in the NUTRIREA2 trial and in the current ancillary study [13]. Thus, the route for artificial nutrition in patients receiving invasive mechanical ventilation should not be determined based on the risk for microaspiration of gastric content and VAP.

In addition to the above-discussed limitations, pepsin and salivary amylase were only measured during 48 h and not during the whole period of mechanical ventilation. In addition, the study was not blinded. However, the measurement of pepsin and amylase was performed in a blinded manner. Further, our definition of abundant microaspiration was stringent, and one could argue that if a different cutoff had been used, our results might have been different. However, similar results were obtained regarding the percentage of tracheal aspirates positive for pepsin between the two groups. Strengths of our study include the randomized controlled multicenter design, the use of quantitative markers of microaspiration, and the relatively large number of patients and tracheal aspirates analyzed.

## Conclusions

Our results suggest that enteral nutrition is associated with a reduced risk for microaspiration of gastric contents and an increased risk for microaspiration of oropharyngeal secretions. The route for nutrition in mechanically ventilated patients with shock should probably not be determined based on the risk of microaspiration and VAP.

## Abbreviations

CI: Confidence interval
OR: Odds ratio
RGV: Residual gastric volume
VAP: Ventilator-associated pneumonia
